# New-Onset Graves’ Disease in the Background of Hashimoto’s Thyroiditis: Spectrums of the Same Disease With Changing Autoantibodies

**DOI:** 10.7759/cureus.28296

**Published:** 2022-08-23

**Authors:** Iqra Arshad, Tasneem Zahra, Julia Vargas-Jerez

**Affiliations:** 1 Internal Medicine, Lincoln Medical Center, New York, USA; 2 Endocrinology and Diabetes, Lincoln Medical Center, New York, USA; 3 Endocrinology, Diabetes and Metabolism, Lincoln Medical Center, New York, USA

**Keywords:** graves’ disease, thyroid-stimulating immunoglobulin (tsi), levothyroxine, autoantibodies switch, anti-tpo antibodies, hashimoto’s thyroiditis

## Abstract

Hashimoto’s thyroiditis and Graves’ disease represent two spectrums of the same autoimmune thyroiditis. Evolution from Graves’ disease to Hashimoto’s thyroiditis is a common scenario but conversion is a rare occurrence that we observed in the presented case of a 56-year-old Hispanic female with a history of Hashimoto’s thyroiditis with positive anti-thyroperoxidase (TPO) antibodies who was euthyroid on levothbefore75 µg for six years prior to her presentation to the emergency department (ED) with complaints of palpitations, exertional dyspnea, and unintentional weight loss of 20 lbs for four months. The patient denied any recent change in levothyroxine dosage. The patient was noted to have tachycardia at rest, hyperdynamic circulation, and fine tremors on outstretched hands-on examination but no exophthalmos, thyromegaly, or pedal swellings. Her initial laboratory workup revealed thyroid-stimulating hormone of <0.01 mIU/L, with elevated free T3 and free T4 levels. Levothyroxine was held and beta-blockade therapy was started for symptom control. Further workup showed elevated thyroid-stimulating immunoglobulin and thyroid receptor antibody levels and normalization of anti-TPO antibody levels. The radioactive iodine uptake scan was initially delayed because the patient underwent a pulmonary angiogram in the ED. A later scan showed thyromegaly with heterogeneous uptake of 82% in both lobes. Hence, the patient was diagnosed with Graves’ disease and managed with radioactive iodine ablation therapy. On follow-up, the patient developed post-ablation hypothyroidism; she was started back on levothyroxine therapy and became euthyroid. This case highlights that patients can develop Graves’ disease in the background of a hypothyroid state, and this conversion might be postulated secondary to a combination of atypical destructive thyroiditis and a switch of autoantibodies from blocking to stimulating ones. Clinicians should suspect the possibility of changing antibodies when there is a change in the patient’s euthyroid state.

## Introduction

Graves’ disease and Hashimoto’s thyroiditis are autoimmune thyroid disorders (ATDs). Hashimoto’s thyroiditis is characterized by the autoimmune destruction of thyroid cells by autoantibodies resulting in hypothyroidism, while Graves’ disease is caused by developing stimulating autoantibodies to thyroid cells (thyroid-stimulating immunoglobulin, TSI) and thyroid-stimulating hormone (TSH) receptors resulting in hyperthyroidism. Whether these two disorders are separate clinical entities or inter-related has been a debate for years; however, the current view is that they fall under the spectrum of autoimmune thyroid disorders and represent the two ends of the spectrum [1]. The evolution from Graves’ disease to Hashimoto’s thyroiditis is a common occurrence and has been reported in approximately 15-20% of patients with Graves’ disease [2]. However, the development of Graves’ disease in the background of Hashimoto’s thyroiditis is rare and the exact incidence of this occurrence is unknown [3]. In the described case, the patient developed Graves’ disease in the background of Hashimoto’s thyroiditis and stable treatment with levothyroxine therapy for six years prior to her presentation.

## Case presentation

**Figure 1:**
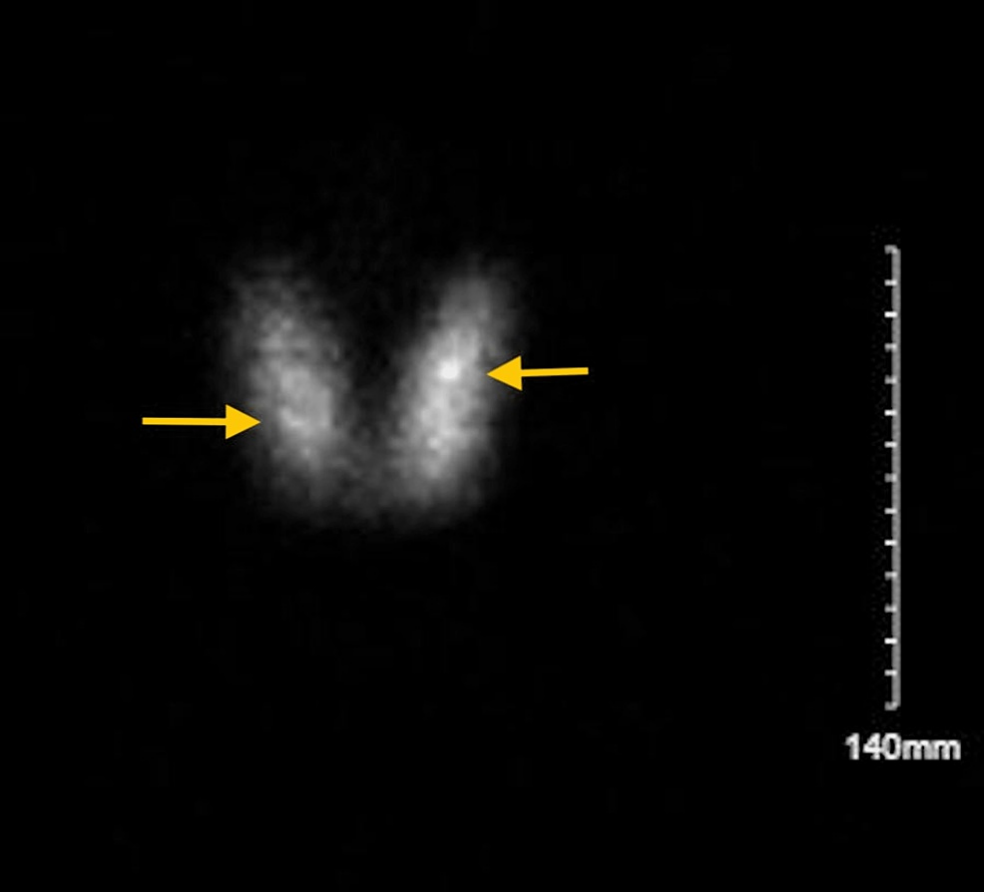
Nuclear medicine uptake scan showing thyromegaly and diffuse uptake in both lobes as shown by arrows.

We present the case of a 56-year-old Hispanic female with a medical history of osteoarthritis, osteopenia, and Hashimoto’s thyroiditis (anti-thyroperoxidase (anti-TPO) antibodies positive) diagnosed six years prior to her presentation. After diagnosis, the patient was initially started on levothyroxine 50 µg daily but did not achieve euthyroidism despite good compliance. The dose was increased to 75 µg daily by her primary medical doctor and the patient became euthyroid. The patient was doing well until her presentation to the Emergency Department (ED) with complaints of palpitations, exertional shortness of breath, and decreased exercise tolerance for the last three to four months. She denied any recent change of brand and dosage of levothyroxine and was compliant with the prescribed dose. In addition to the aforementioned symptoms, she also complained of sleep disturbances, anxious mood, and unintentional weight loss of 20 lbs over the last four months but denied associated chest and abdominal pain and altered bowel habits. Physical examination was notable for tachycardia to 120-130s at rest, hyperdynamic precordium with non-displaced heaving apex beat, normal S1 and S2 with no murmurs/gallops/rubs, visible pulsations in neck bilaterally, bounding pulses throughout extremities, and tremors on outstretched hands, but negative for exophthalmos, thyromegaly, and pedal edema. A 12-lead electrocardiogram (ECG) showed sinus tachycardia. The patient received intravenous fluid resuscitation in the ED with no improvement of tachycardia. Computerized tomography (CT) pulmonary angiogram was done in the ED and pulmonary embolism was ruled out. The patient was admitted to the medical floor for further management. Initial laboratory workup showed TSH of <0.01 mIU/L (0.2-4.2 mIU/L), free T3 of 14.50 pg/mL (1.8-4.6 pg/mL) and free T4 of 5.5 ng/dL (0.9-1.8 ng/dL). Levothyroxine was held and the patient was started on atenolol 25 mg for symptom control. Further workup revealed positive TSI with levels of 5.69 IU/L (0-0.55 IU/L), thyroid receptor antibodies (TRAb) with levels of 8.14 IU/L (0.0-1.75 IU/L), and normal anti-TPO antibodies with levels of 20.1 IU/mL (≤34.9 IU/mL). A prior small right upper lobe nodule was noted to decrease in size and no new nodules were found on repeat thyroid ultrasound. The nuclear medicine (NM) uptake scan was delayed up to six weeks because the patient received iodinated contrast in ED. The patient was followed up in the clinic and later uptake scan showed thyromegaly with heterogeneous uptake of 82% in both lobes in 24 hours, highly suggestive of Graves’ disease (Figure 1).

The patient was diagnosed with Graves’ disease. Treatment options, including antithyroid medications versus radioactive iodine ablation therapy, were discussed with the patient. The patient opted for ablation therapy. Post-ablation, symptoms resolved completely. Atenolol was discontinued. Free T3 and T4 returned to normal limits. The patient developed post-ablation hypothyroidism with a TSH of 62 and was started again on levothyroxine therapy with both clinical and biochemical improvement. The patient is euthyroid to date and regularly follows up with our endocrine clinic.

## Discussion

In literature, different mechanisms have been proposed for the conversion of Hashimoto’s thyroiditis to Graves’ disease, including, but not limited to, a combination of immune-mediated destruction of thyroid cells and development of Graves’ disease-associated autoantibodies [4]. The authors have further hypothesized the mechanism responsible for this conversion as a switch of autoantibodies from thyrotropin receptor-blocking antibodies (TBAb) to thyrotropin receptor (TRAb) and thyroid-stimulating antibodies (TSAb) resulting in hyperthyroidism [4]. We observed similar findings in our case where the patient developed predominantly stimulating autoantibodies (TSI and TRAb) with normalization of thyroperoxidase (TPO) antibodies in the background of her hypothyroid state secondary to Hashimoto’s thyroiditis. Factors that could affect this switch of autoantibodies include treatment with levothyroxine therapy which is usually associated with suppression of autoantibodies but in unusual cases can either induce or enhance the autoantibody levels; treatment with antithyroid drugs, immunosuppressed state, and dysregulation of CD4+ helper T-cells, which result in loss of control over autoimmunity and paradigm shift from blocking to stimulating antibodies. In our case, the factor that might have contributed to this switch could be the levothyroxine therapy that patient was taking before developing full-blown Graves’ disease. It is pertinent to know that levothyroxine therapy could either increase the ongoing action of stimulating autoantibodies or induce their production in patients who may or may not have TBAb at baseline [5]. The mechanism behind this could be that the rise in serum-free T4 levels from levothyroxine therapy causes an increased expression of stimulatory molecules on thyroid cells that stimulates antibody production. Alternatively, the activity of blocking antibodies falls below the activity of stimulating ones, resulting in a switch [5]. In a case series on the development of Graves’ disease after painful and painless thyroiditis, the authors proposed that damage to thyroid cells itself could act as a trigger and lead to the development of TSH receptor antibodies which changed their effects from blocking to stimulating ones to produce hyperthyroid state [6-8]. In the literature, an older proposed mechanism was that damage to thyroid epithelial cells causes thyroid hormone leakage, which, in turn, stimulates microsomal antigens and subsequently induces helper T cells to produce thyroid-stimulating antibodies [7].

In a case report by Clifford and Wakil where the patient developed severe Graves disease from primary subclinical hypothyroidism and treatment with levothyroxine therapy for six years. However, contrary to our case, the clinical course was complicated with agranulocytosis from antithyroid medications. Later, the patient’s hyperthyroidism was managed surgically with thyroidectomy [9].

One of the weaknesses in our case was that the patient was not checked for thyroid receptor antibodies (TRAb) that could be stimulating or blocking at the time of diagnosis of hypothyroidism, and was started on levothyroxine by her primary doctor as she met the clinical and biochemical criteria. There is evidence that in some cases of Graves’ disease, patients may develop a preceding hypothyroid state with positive TRAb. We could not rule out this possibility in our case; however, later development of a clinical hyperthyroid state with positive thyroid-stimulating antibodies (TSI and TRAb) and a diffuse uptake on nuclear medicine scan suggest that her evolution from Hashimoto’s thyroiditis to Graves’ disease could be secondary to a switch of autoantibodies that might have been potentiated by levothyroxine treatment in her case.

## Conclusions

There is strong clinical evidence to support the autoimmune origin of Graves’ disease but data to support its evolution from Hashimoto’s thyroiditis is scarce. In most cases, this occurrence is often under-reported. Clinicians should suspect the switch of autoantibodies if there is any clinical or biochemical evidence of change in the patient’s euthyroid state. A thorough evaluation should be done so that timely and appropriate management can be offered. Further immunological and genetic studies are needed to explain this unusual autoimmune change.
